# *N*-acetylglucosamine drives myelination by triggering oligodendrocyte precursor cell differentiation

**DOI:** 10.1074/jbc.RA120.015595

**Published:** 2020-09-25

**Authors:** Michael Sy, Alexander U. Brandt, Sung-Uk Lee, Barbara L. Newton, Judy Pawling, Autreen Golzar, Anas M. A. Rahman, Zhaoxia Yu, Graham Cooper, Michael Scheel, Friedemann Paul, James W. Dennis, Michael Demetriou

**Affiliations:** 1 Department of Neurology, University of California Irvine, Irvine, California, USA; 2 Experimental and Clinical Research Center, Charité – Universitätsmedizin Berlin and Max-Delbrueck-Center for Molecular Medicine, Berlin, Germany; 3 NeuroCure Clinical Research Center, Charité – Universitätsmedizin Berlin, corporate member of Freie Universität Berlin, Humboldt-Universität zu Berlin and Berlin Institute of Health, Berlin, Germany; 4 Samuel Lunenfeld Research Institute, Mount Sinai Hospital, Toronto, Canada; 5 Department of Microbiology and Molecular Genetics, University of California Irvine, Irvine, California, USA; 6 Department of Molecular Genetics, University of Toronto, Toronto, Canada; 7 Department of Statistics, Donald Bren School of Information and Computer Sciences, University of California Irvine, Irvine, California, USA; 8 Einstein Center for Neurosciences, Berlin, Germany; 9 Department of Experimental Neurology and Center for Stroke Research, Berlin, Charité – Universitätsmedizin Berlin, Berlin, Germany

**Keywords:** *N*-glycan branching, *N*-acetylglucosamine, oligodendrocytes, myelination, myelin repair, multiple sclerosis, *N*-linked glycosylation, oligodendrocyte, myelin, metabolism, oligodendrocyte precursor cell

## Abstract

Myelination plays an important role in cognitive development and in demyelinating diseases like multiple sclerosis (MS), where failure of remyelination promotes permanent neuro-axonal damage. Modification of cell surface receptors with branched *N*-glycans coordinates cell growth and differentiation by controlling glycoprotein clustering, signaling, and endocytosis. GlcNAc is a rate-limiting metabolite for *N*-glycan branching. Here we report that GlcNAc and *N*-glycan branching trigger oligodendrogenesis from precursor cells by inhibiting platelet-derived growth factor receptor-α cell endocytosis. Supplying oral GlcNAc to lactating mice drives primary myelination in newborn pups via secretion in breast milk, whereas genetically blocking *N*-glycan branching markedly inhibits primary myelination. In adult mice with toxin (cuprizone)-induced demyelination, oral GlcNAc prevents neuro-axonal damage by driving myelin repair. In MS patients, endogenous serum GlcNAc levels inversely correlated with imaging measures of demyelination and microstructural damage. Our data identify *N*-glycan branching and GlcNAc as critical regulators of primary myelination and myelin repair and suggest that oral GlcNAc may be neuroprotective in demyelinating diseases like MS.

Myelination of axons by oligodendrocytes in the central nervous system plays a critical role in normal cognitive development and function and in demyelinating disease such as multiple sclerosis (MS) (1, 2). In addition to speeding conduction of the action potential, myelination supports axon health and survival (3–5). In MS, remyelination of demyelinated axons by oligodendrocytes is often incomplete despite the presence of abundant oligodendrocyte precursor cells (OPC) throughout the brain (6–10). The molecular mechanisms that block remyelination in MS are incompletely understood, and there is a lack of therapies to promote myelin repair. Failure to adequately remyelinate is influenced by the microenvironment of the MS lesion, where reactive astrocytes, microglia, and macrophages produce various inhibitory factors leading to disruption in OPC differentiation, oligodendrocyte migration, process outgrowth, and attachment to axons (11). Multiple studies have identified molecules that limit OPC differentiation into myelin-producing cells including LINGO-1 (12), various extracellular matrix proteins (13, 14), and myelin debris (15). Thus, increasing OPC differentiation has become an important strategy for promoting remyelination in MS and other demyelinating diseases (16).

Cell surface and secreted proteins are co- and post-translationally modified on Asn(*N*) by the addition of carbohydrates (*N*-glycans) in the endoplasmic reticulum and subsequently remodeled in the Golgi. The degree of GlcNAc branching in *N*-glycans promotes binding to galectins, a family of sugar-binding proteins (Fig. S1*A*). Polyvalent galectin-glycoprotein interactions at the cell surface form a macromolecular lattice that simultaneously controls the movement, clustering, and/or endocytosis of multiple receptors and transporters to control signaling, cell growth, differentiation, and death (17–24). For example, *N*-glycan branching controls epithelial cell growth by regulating receptor tyrosine kinases endocytosis (17–20), promotes glucose uptake in mesenchymal and pancreatic β cells by inhibiting glucose transporter endocytosis (19, 25), and reduces T-cell, B-cell, and neutrophil pro-inflammatory responses by coregulating the clustering and/or endocytosis of multiple glycoproteins (17, 21, 23, 27–29). These mechanisms in turn impact cancer, type II diabetes, and autoimmunity (20). For example, reductions in *N*-glycan branching are associated with MS and promote both inflammatory demyelination and neurodegeneration in mice, the latter by an unknown mechanism (17, 30–36).

**Figure 1.**
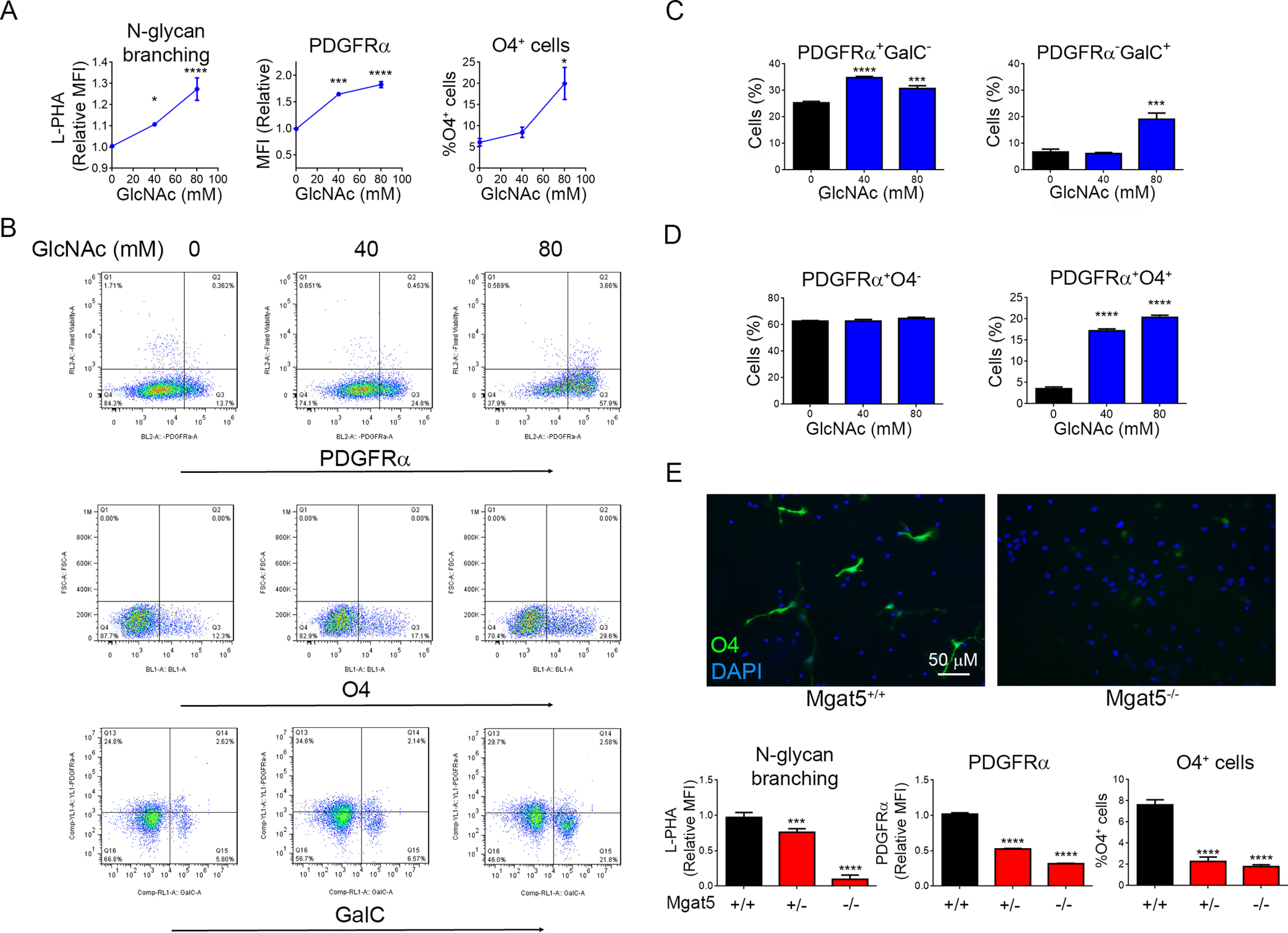
**GlcNAc and *N*-glycan branching promotes oligodendrogenesis.** *A*–*D,* flow cytometry of E12.5 NSCs from CD1 (*B* and *C*) or C57BL/6 (*D*) mice cultured in growth media (FGF + EGF) ± GlcNAc for 48 h. Cell surface binding levels of L-PHA and PDGFRα are measured as mean fluorescence intensity (*MFI*). *E*, flow cytometry and immunofluorescence microscopy of E12.5 NSCs in differentiation media (FGF + PDGF-AA) from *Mgat5*^+/+^, *Mgat5*^+/−^, and *Mgat5*^−/−^ C57BL/6 mice. Data are three technical replicates per group (*A*–*E*), representative of 3 (*A*–*C*) or two (*D* and *E*) experiments. *p*-values are by one-way ANOVA with Sidak's multiple comparison test. All *error bars* are standard error. *p < 0.05, **p < 0.01, ***p < 0.001, ****p < 0.0001.

Given the diverse and pleiotropic effects of *N*-glycan branching, identifying and manipulating regulatory mechanisms may provide new insights into disease pathogenesis and opportunities for therapeutic intervention. In this regard, metabolism is a critical regulator of *N*-glycan branching by controlling availability of the sugar-nucleotide UDP-GlcNAc, the substrate used by the Mgat family of *N*-glycan branching enzymes (19, 20, 24, 37, 38). UDP-GlcNAc is generated in the hexosamine pathway *de novo* from glucose or by salvage from GlcNAc. Extracellular GlcNAc enters cells through micropinocytosis, with supplementation of cells or mice with GlcNAc inhibiting pro-inflammatory T-cell responses and murine models of inflammatory demyelination by enhancing *N*-glycan branching (19, 31, 37, 38).

Targeted deletion of galectin-3, a ligand for *N*-glycan branching, leads to decreased production of oligodendrocytes, poor myelination of axons, and reduced ability to remyelinate after injury (39). In humans, loss-of-function mutations in PGM3, a gene required to generate branched *N*-glycans from GlcNAc, display reduced branching and severe CNS hypomyelination (40). Platelet-derived growth factor–AA plays a critical role in oligodendrogenesis (41), with its receptor (PDGFRα) expressed in oligodendrocyte progenitor/precursor cells (42). In epithelial cells, *N*-glycan branching deficiency reduces PDGFRα surface expression by enhancing loss via endocytosis, leading to reduced signaling (18). Thus, here we examine the hypothesis that GlcNAc may provide an oral therapeutic to raise *N*-glycan branching in OPCs, promote myelination, and reduce the potential for neurodegeneration by initiating oligodendrocyte differentiation via enhanced PDGFRα surface expression and signaling in OPCs.

## Results

### GlcNAc and N-glycan branching trigger oligodendrogenesis by inhibiting PDGFRα endocytosis

We examined oligodendrogenesis *in vitro* using mouse neural stem cells (NSC) derived from the medial ganglionic eminence of E12.5 mouse embryos, where OPCs first appear. GlcNAc treatment of NSCs for 48 h in growth media lacking exogenous differentiation cytokines (*i.e.* no PDGF-AA, T3, or CNTF) significantly increased *N*-glycan branching and PDGFRα surface expression (Fig. 1, *A* and *B*), the former assessed by L-PHA (*Phaseolus vulgaris* leukoagglutinin) flow cytometry (17, 22). Consistent with increased PDGFRα surface expression, GlcNAc also promoted pre-oligodendrocyte differentiation as evidenced by augmented expression of oligodendrocyte transcription factor (OLIG2) and increased numbers of O4- and GalC-positive cells (Fig. 1, *A***–***C* and Fig. S1, *B* and *C*). Double staining for PDGFRα and O4 revealed that GlcNAc promoted development of pre-oligodendrocytes (PDGFRα^+^O4^+^) with no change in the number of OPCs (PDGFRα^+^O4^−^) (Fig. 1*D*). Thus, after only 2 days of culture, GlcNAc initiated oligodendrogenesis from NSCs despite the absence of exogenous differentiation cytokines such as PDGF-AA. Remarkably, GlcNAc in growth media was more potent than differentiation media containing exogenous PDGF-AA at initiating oligodendrogenesis (Fig. S1*D*). Combining GlcNAc with PDGF-AA also enhanced NSC differentiation to O4^+^ pre-oligodendrocytes (Fig. S1*E*).

To confirm a role for *N*-glycan branching in oligodendrogenesis, we first used kifunensine to inhibit *N*-glycan branching (23) in NSC induced to differentiate by exogenous PDGF-AA. Reducing branching in NSCs using kifunensine significantly reduced PDGFRα surface expression and the number of O4^+^ cells induced by PDGF-AA differentiation media (Fig. S1*F*). To confirm this result genetically, we utilized *Mgat5*^−/−^ and doxycycline-inducible *Mgat1^f/f^/tetO-cre/ROSA-rtTA* mice (17, 22). *Mgat5* deletion mildly reduces *N*-glycan branching, whereas *Mgat1* deletion completely blocks *N*-glycan branching (Fig. S1*A*). *In vitro* doxycycline treatment of NSC from *Mgat1^f/f^/tetO-cre/ROSA-rtTA* mice readily induced deletion of *Mgat1*, as measured by loss of L-PHA binding (Fig. S1*G*). *Mgat5*- and *Mgat1*-deleted NSCs displayed decreased surface levels of PDGFRα and were markedly reduced in their ability to differentiate into O4^+^ pre-oligodendrocytes in response to PDGF-AA differentiation media (Fig. 1*E* and Fig. S1*G*). In *Mgat5* heterozygous NSCs, small reductions in *N*-glycan branching also inhibited oligodendrocyte differentiation (Fig. 1*E*). Thus, subtle changes in *N*-glycan branching can markedly impact oligodendrocyte differentiation from NSC *in vitro*.

### GlcNAc and N-glycan branching promote primary myelination in mice

**Figure 2.**
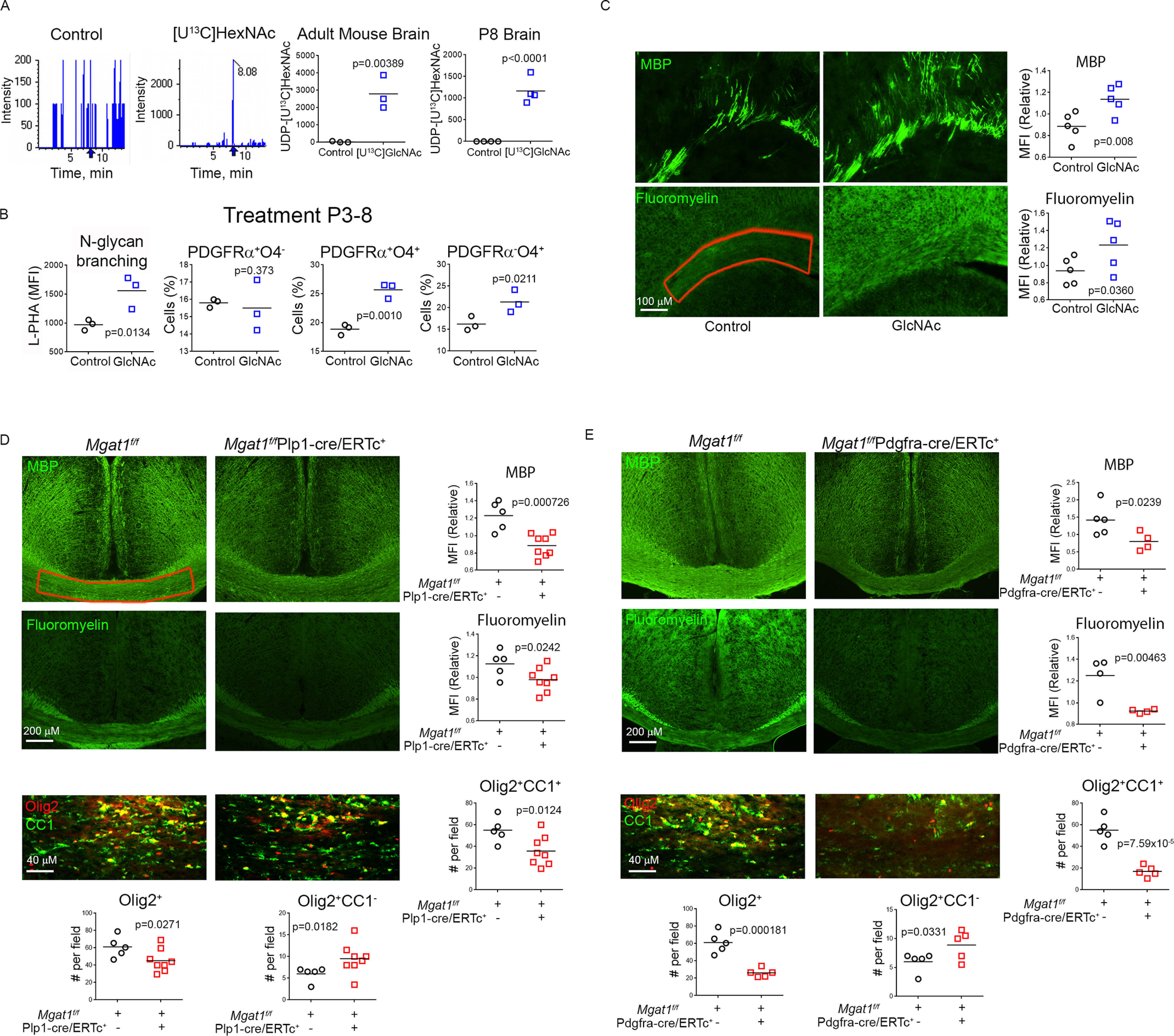
**GlcNAc and *N*-glycan branching promote primary myelination.** *A*–*C*, newborn PL/J mice were given exogenous GlcNAc or [U^13^C]GlcNAc by providing their nursing mothers with GlcNAc or [U^13^C]GlcNAc at 1 mg/ml in drinking water from P3–P8. The pups and mothers were sacrificed at P8 and brains were analyzed by LC–MS/MS for UDP-[U^13^C]HexNAc (*A*, *n* = 3, 3 adult, *n* = 4,4 P8 pups), flow cytometry (*B*, *n* = 3, 3) or immunofluorescence microscopy (*C*, *n* = 5,5) for the indicated markers. L-PHA staining in (*B*) is gated on PDGFRα^+^ cells. Data in (*C*) is the average of fluorescence intensity of the area depicted in *red* of three brain slices per mouse. One-sided *t* test. *D* and *E*, the indicated adult mice (10 weeks old) were treated with tamoxifen at weeks 0 and 4 and sacrificed at week 8, and brains were analyzed by immunofluorescence microscopy for MBP, myelin (FluoroMyelin), Olig2^+^, and CC1^+^ cells (*n* = 5 (2 male, 3 female), 8 (6 male, 2 female) (*D*), and *n* = 5 (2 male, 3 female), 4 (2 male, 2 female) (*E*); one-sided *t* test). Each data point in the graphs represents average fluorescence or cell counts of the highlighted area from three (*D*) or two (*E*) different brain slices per mouse.

Next, we examined whether oral GlcNAc can cross the blood-brain barrier to promote oligodendrocyte differentiation and myelination *in vivo*. Adult mice (*n* = 6) and lactating mothers were provided with/without ^13^C-labeled GlcNAc ([U^13^C]GlcNAc) (10) in their drinking water, and metabolites derived from perfused brains were analyzed by liquid chromatography–tandem mass spectrometry (LC–MS/MS). Although this method does not resolve stereoisomers of *N*-acetylhexosamines (*i.e.* GlcNAc *versus* GalNAc), a reversible 4-epimerase equilibrates UDP-GlcNAc and UDP-GalNAc *in vivo* (19). LC–MS/MS identified UDP-[U^13^C]-*N*-acetylhexosamines (UDP-[U^13^C]-HexNAc) in treated adult female mouse brains and in the brains of their suckling pups (Fig. 2*A*). This demonstrates that orally delivered GlcNAc is not only able to cross the blood-brain barrier and be metabolized to UDP-GlcNAc by CNS cells but is also secreted at sufficient levels in breast milk to raise UDP-GlcNAc in the brains of suckling pups.

To assess whether oral GlcNAc promotes oligodendrogenesis *in vivo* in the absence of inflammation, we examined primary myelination in mice during the early perinatal period. We provided GlcNAc or vehicle to pregnant/lactating female mice from E12.5, postnatal day 3 (P3) or P5 through to P8. Indeed, oral GlcNAc increased *N*-glycan branching in PDGFRα^+^ cells and the number of pre-oligodendrocytes (PDGFRα^+^O4^+^), immature oligodendrocytes (PDGFRα^−^O4^+^), and mature oligodendrocytes (MBP^+^), with little effect on the number of OPCs (PDGFRα^+^O4^−^) (Fig. 2*B* and Fig. S2*A*). The lack of change in OPC number is consistent with our *in vitro* data (Fig. 1*D*) and suggests that GlcNAc promotes OPC self-renewal and/or NSC differentiation to OPC, resulting in a stable number of OPCs. Consistent with increased oligodendrogenesis, oral GlcNAc also increased primary myelination when provided to pups from P3-8, as assessed by increased levels of staining for myelin basic protein (MBP) and myelin (as measured by FluoroMyelin) (Fig. 2*C*).

To confirm that *N*-glycan branching promotes myelination in the absence of inflammation *in vivo*, we generated mice with tamoxifen-inducible deletion of *Mgat1* only in OPCs and oligodendrocytes, namely *Mgat1^f/f^*Plp1-cre/ERTc^+^ mice. Because proteolipid protein (PLP) promoter–driven Cre expression only becomes restricted to the oligodendrocyte lineage (OPC and oligodendrocyte) at P28 (43), we focused on adult mice. OPCs continue to proliferate and generate significant new myelin in adulthood, with myelination gradually doubling from ∼2–10 months (44–46). Tamoxifen readily induced *Mgat1* deletion in O4^+^ oligodendrocytes but not O4^−^ cells *in vivo*, as determined by loss of L-PHA binding by flow cytometry (Fig. S2*B*). Consistent with slow accumulation of new myelin from OPCs during adulthood, 2 weeks following tamoxifen treatment (*Mgat1* deletion) adult *Mgat1^f/f^*Plp1-cre/ERTc^+^ mice did not display significant differences in brain levels of MBP or myelin (FluoroMyelin) relative to tamoxifen-treated controls (Fig. S2*C*). However, 8 weeks after initial tamoxifen treatment, *Mgat1* deletion resulted in significant reductions in levels of MBP, myelin (FluoroMyelin), and number of total (Olig2^+^) and mature (Olig2^+^CC1^+^) oligodendrocytes, along with increased numbers of immature (Olig2^+^CC1^−^) oligodendrocytes (Fig. 2*D* and Fig. S2*D*). To confirm that these results primarily arose from a defect in new myelin formation from OPCs, rather than a defect in mature oligodendrocytes, we generated *Mgat1^f/f^*Pdgfra-creER^+^ mice where tamoxifen induces deletion of *Mgat1* in OPCs but not mature oligodendrocytes. Indeed, 8 weeks but not 2 weeks after tamoxifen treatment, deletion of *Mgat1* in OPCs significantly reduced levels of MBP, myelin (FluoroMyelin), and number of total (Olig2^+^) and mature (Olig2^+^CC1^+^) oligodendrocytes along with increased numbers of immature (Olig2^+^CC1^−^) oligodendrocytes (Fig. 2*E* and Fig. S2*E*). Tamoxifen has been reported to promote myelination (47); however, *Mgat1* deletion reduced myelination despite potential positive effects of tamoxifen. Together, these data demonstrate that GlcNAc and *N*-glycan branching promote primary myelination in mice by driving OPC differentiation.

### GlcNAc prevents damage to demyelinated axons by promoting myelin repair

**Figure 3.**
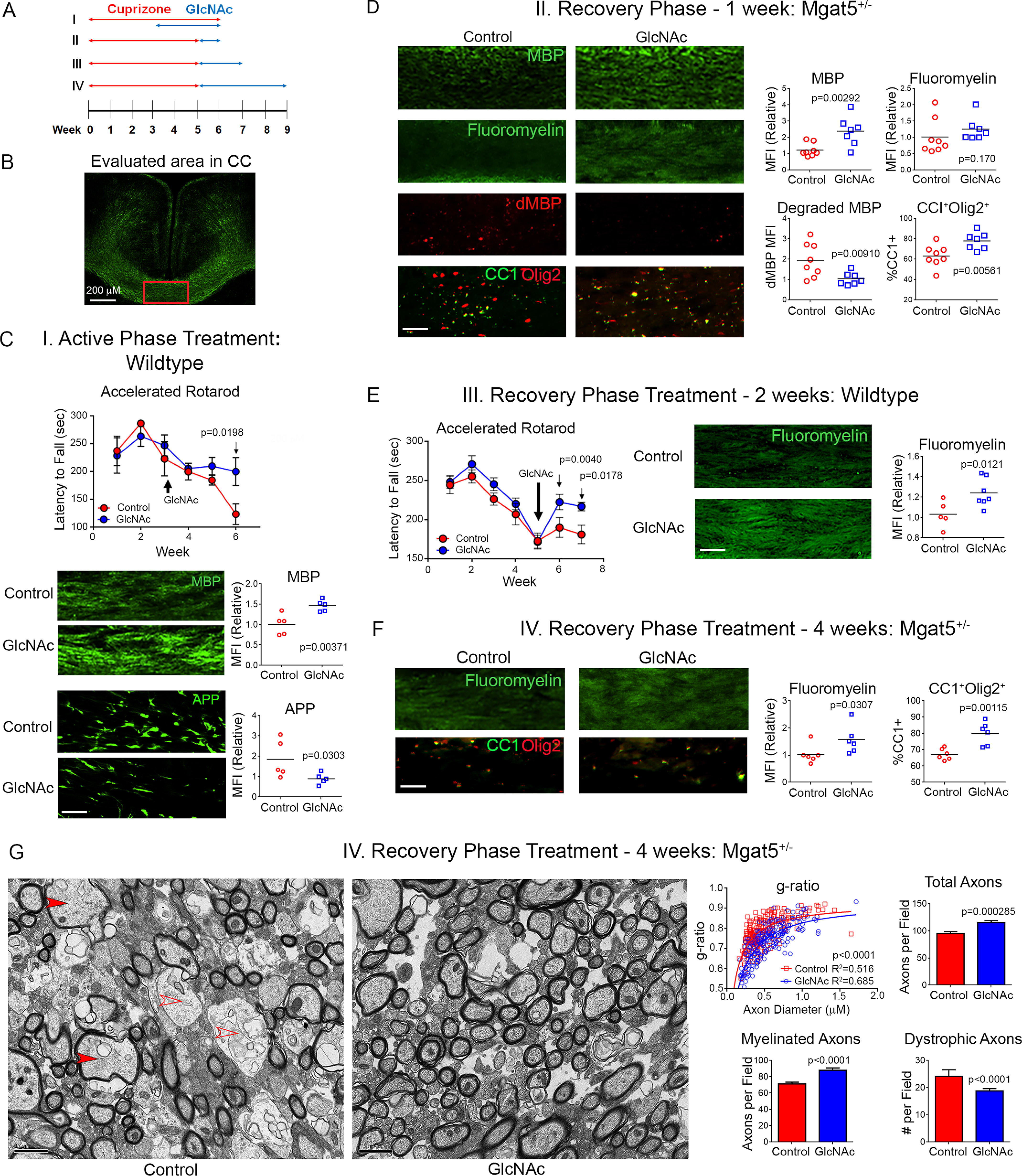
**Oral GlcNAc promotes remyelination and limits axonal injury.** *A*, the ability of GlcNAc to promote myelin repair *in vivo* was assessed using the cuprizone model, with oral GlcNAc treatment (1 mg/ml in drinking water) during the last 3 weeks of a 6-week cuprizone exposure (I, active phase treatment) or after 5 weeks of cuprizone treatment for 1, 2, or 4 weeks (II, III, and IV, recovery phase treatment). WT (I and III) or *Mgat5* heterozygous (II and IV) C57BL/6 mice were used. *B*, area of the medial corpus callosum (*CC*) analyzed. *C*–*F*, shown is the latency to fall in an accelerated rotarod test and immunofluorescence staining of the corpus callosum for MBP, degraded MBP (*dMBP*), APP, myelin (FluoroMyelin), and/or CC1/Olig2 from WT mice with active phase GlcNAc treatment (C, *n* = 5,5, all male), *Mgat5* heterozygous mice with recovery phase GlcNAc treatment for 1 week (*D*, *n* = 8 (5 male, 3 female), 7 (4 male, 3 female)), WT mice with recovery phase GlcNAc treatment for 2 weeks (*E*, *n* = 11,14 for rotarod, *n* = 5,7 for immunofluorescence, all male), or *Mgat5* heterozygous mice with active phase GlcNAc for 4 weeks (*F*, *n* = 6,6 with 4 male and 2 female per group). Data points represents average fluorescence from 3–4 different brain slices per mouse. Rotarod *p*-values by 2-way ANOVA with Sidak's multiple comparisons post-test. Immunofluorescence *p*-values by one-tailed *t* test. *Scale bar* = 50 µm. *G*, the CC of mice from the 4-week recovery phase treatment group in panel (*F*) were analyzed by EM (*n* = 3,3 with 2 male and 1 female per group). Representative electron micrographs in control and GlcNAc treatment groups are shown, *scale bar* = 1 µm. *Filled* and *empty arrowheads* indicate examples of myelinated and unmyelinated dystrophic axons, respectively. Plot of g-ratio *versus* axon diameter (*n* = 214 and 222 axons) was counted blindly from two fields (105µm^2^) per mouse (*p*-value comparing best fit curves from nonlinear regression, R^2^ is the goodness of fit for each group). Numbers of total axons, myelinated axons, and dystrophic axons (axon diameter > 0.7 µm) were counted blindly in six fields (105µm^2^) per mouse in each treatment group (*n* = 18, 18, *p*-value by one-sided *t* test). All *error bars* are standard error.

To explore whether GlcNAc can promote remyelination in adult mice following myelin injury, we utilized the cuprizone model of nonimmune induced demyelination/remyelination on *Mgat5*^+/−^ and WT C57BL/6 mice. Cuprizone at 0.2% induces demyelination in the corpus callosum by 3 weeks, with maximum demyelination at 5–6 weeks. Partial remyelination via maturation of OPCs begins at the height of demyelination and becomes complete ∼3–5 weeks after cuprizone withdrawal. Given this, we examined four different treatment regimens (Fig. 3*A*). When GlcNAc was concurrently provided during the final 3 weeks of a 6-week cuprizone (0.2%) exposure in WT mice, GlcNAc prevented loss of motor function (as measured using rotarod fall latency) while increasing MBP levels and reducing axonal damage (as measured by reduced accumulation of amyloid precursor protein (APP)) in the corpus callosum (Fig. 3*C*). To address potential confounding effects of GlcNAc on inhibiting demyelination by cuprizone during concurrent treatment, we initiated treatment of WT mice for 2 weeks or *Mgat5*^+/−^ mice for 1 or 4 weeks of GlcNAc only after cuprizone was stopped (Fig. 3*A*). This revealed that GlcNAc enhanced levels of MBP, myelin (FluoroMyelin), and mature oligodendrocytes (CC1^+^Olig2^+^) while reducing the amount of degraded MBP (dMBP)/myelin degeneration within the corpus callosum (Fig. 3, *D***–***F*). dMBP was detected by an antibody that specifically recognizes areas of myelin degeneration (48). EM analysis confirmed these results, revealing that GlcNAc enhanced the number of myelinated axons and the degree of myelination as measured by the g-ratio while also reducing axon loss and the number of degenerating and dystrophic/swollen axons (Fig. 3*G* and Fig. S3*A*). GlcNAc also enhanced the number of paranodes, which increase with remyelination (Fig. S3*A*) (49). Enhancement of myelination by GlcNAc depends on time, because the increase in FluoroMyelin staining in *Mgat5*^+/−^ mice was ∼2-fold greater with 4 weeks *versus* 1 week of GlcNAc treatment (Fig. 3, *D* and *F*). Importantly, the subtle reductions in *N*-glycan branching induced in *Mgat5*^+/−^ mice did not alter baseline levels of myelin, yet they reduced remyelination following cuprizone-induced injury relative to *Mgat5*^+/+^ control mice (Fig. S3, *B* and *C*). Together, these data indicate that GlcNAc and *N*-glycan branching promote myelin repair and provide neuro-protection to axons following demyelination.

### A marker of serum GlcNAc inversely associates with imaging markers of myelin-axon damage

**Figure 4.**
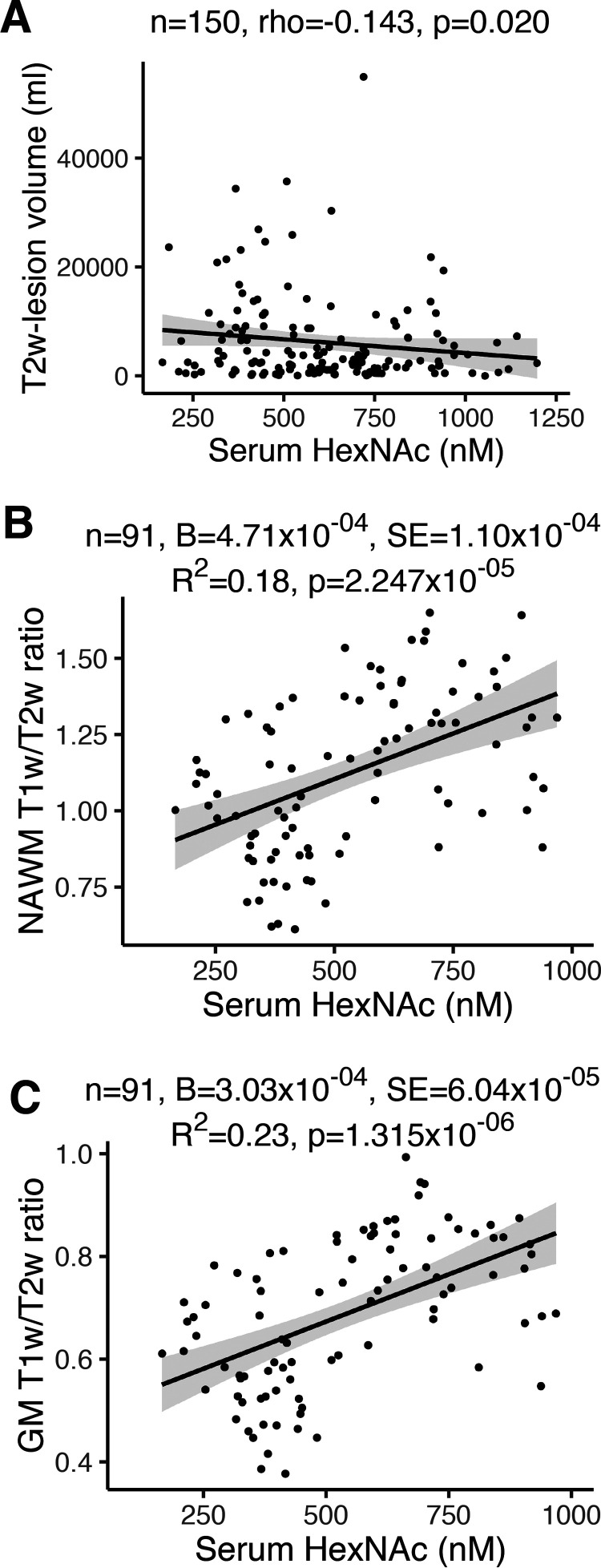
**Serum HexNAc correlates with markers of myelin-axon microstructural damage in MS patients.** *A*–*C*, association of serum HexNAc levels with MRI measures of myelin-axon microstructural damage in a cohort of *n* = 180 MS patients. T2w lesion volume (*A*) and T1w/T2w ratio in normal-appearing white matter (*NAWM*, *B*) and gray matter (*GM*, *C*) is shown. Coefficient B, standard error (S.E.), and R^2^ are from linear regression models correcting for age and sex (in *B* and *C*). *Black lines* in regression models represent coefficients from noncorrected models, *gray areas* show the 95% confidence interval. Value in nM are serum HexNAc levels.

To explore whether alterations in GlcNAc may impact myelination status in MS patients, we used a cohort of 180 MS patients to correlate endogenous serum HexNAc levels with measures of white matter damage by MRI of the brain. Increased T2w lesion volume and count on brain MRI are measures of the extent and frequency of demyelination, respectively. T2w lesion volume correlated with lower HexNAc serum levels (Fig. 4*A*, *p* = 0.020), whereas T2w lesion count did not (*p* = 0.387). Likewise, patients with contrast-enhancing lesions, a marker of active inflammation in MS, had similar serum HexNAc levels to those without (*p* = 0.866), suggesting that GlcNAc primarily affects the extent of permanent demyelination rather than initiation of inflammatory demyelination. T1w/T2w ratio maps (50) reflect microstructural integrity of myelin/axons in normal-appearing white matter (51) and cortical gray matter (52, 53). With age and gender as covariates, low serum HexNAc levels were strongly associated with lower T1w/T2w ratios indicating microstructural damage of myelin/axons in both normal-appearing white matter (r^2^ = 0.18, *p* = 2.25 × 10^−5^) and gray matter (r^2^ = 0.23, *p* = 1.32 × 10^−6^), (Fig. 4, *B* and *C*). Together, these data are consistent with our mouse data and suggest that GlcNAc may promote myelination in MS.

## Discussion

Here we report a novel pathway for regulating oligodendrogenesis, primary myelination, and myelin repair by *N*-glycan branching and GlcNAc. Our data demonstrate that GlcNAc and *N*-glycan branching are neuroprotective for demyelinated axons by promoting oligodendrogenesis and myelination from OPCs. The association of low endogenous GlcNAc with increased myelin-axon microstructural damage in MS patients suggests that this mechanism is relevant to pathogenesis of MS. This hypothesis is consistent with recent data suggesting that some MS patients are blocked in their ability to generate new myelin from progenitors (9). The mechanisms that drive neurodegeneration in MS are poorly understood, and our data raise the possibility that alterations in *N*-glycan branching and/or GlcNAc availability may promote neurodegeneration by blocking remyelination. Indeed, we find that low levels of serum GlcNAc in MS patients is associated with a progressive disease course, clinical disability, and multiple neuroimaging measures of neurodegeneration (unpublished data).

Providing oral GlcNAc to lactating female mice increased primary myelination in nursing pups via delivery of GlcNAc in breast milk. In humans, GlcNAc is a major component of breast milk oligosaccharides (∼1.5 to ∼0.6 mg/ml from term to 13 weeks) (54) and can be released as a monosaccharide by infant microbiota (55). Thus, breastfed newborns consume ∼0.5–1.5 g of GlcNAc per day or ∼100–300 mg/kg/day for a 5-kg infant. This is similar to the ∼160 mg/kg/day dose that we observed to promote myelination in adult mice. In contrast to human breast milk, GlcNAc is not a significant component of commercial baby formula. Breastfed infants display increased myelination and cognitive function compared with formula-fed infants (56, 57), but the mechanism is unknown. Our data suggest that GlcNAc in human breast milk may be a major driver of this effect.

GlcNAc and *N*-glycan branching markedly enhanced cell surface expression of PDGFRα, a critical initiator of OPC differentiation. However, GlcNAc and *N*-glycan branching likely affect other cell surface receptors/transporters in OPCs to drive myelination and promote axonal health. For example, cell motility is significantly enhanced by *N*-glycan branching via reduced clustering of integrins (58, 59). Such activity in OPCs would enhance their ability to traffic to sites of demyelination and promote myelin repair. *N*-glycan branching also stimulates glucose transporter surface retention to enhance glucose uptake (19, 25). Glucose transporter 1 (GLUT1) in oligodendrocytes promotes axonal health and function by increasing transfer of lactate to axons via increased glucose supply to the glycolytic pathway in oligodendrocytes (60). Thus, part of the neuroprotective effect of GlcNAc following myelin repair may be through enhanced transport of glucose into oligodendrocytes.

GlcNAc and/or *N*-glycan branching also play important roles in suppressing T cell– and B cell–mediated inflammatory demyelination (17, 28, 30–33, 35, 37, 38). In T cells, GlcNAc and *N*-glycan branching suppress activation signaling via the T-cell receptor (17, 21, 22), inhibit pro-inflammatory T_H_1 and T_H_17 differentiation (24, 38), and enhance anti-inflammatory T regulatory cell differentiation (24). B-cell depletion is a potent therapy in MS, predominantly acting by suppressing innate antigen presenting cell function rather than via antibody production (61, 62). N-glycan branching in B cells reduces pro-inflammatory innate signaling via toll-like receptors and inhibits antigen presenting cell activity, yet it promotes adaptive responses through the B-cell receptor (28). Thus, oral GlcNAc is uniquely positioned as a therapeutic to reverse three major targets driving MS pathogenesis, namely pro-inflammatory T-cell responses, pro-inflammatory innate B-cell activity, and myelin repair. No current MS therapy has such diverse mechanisms of action.

Concentrations of GlcNAc required to raise *N*-glycan branching *in vivo* are markedly lower than those required for *in vitro* activity (37, 38). This is largely driven by GlcNAc entering cells by macropinocytosis, and therefore both time and rate of membrane turnover can influence concentrations of GlcNAc required to raise *N*-glycan branching (37, 38). Thus, short-term *in vitro* experiments require high concentrations to raise intracellular GlcNAc levels quickly, whereas primary cells remain viable. In contrast, cells can be exposed to GlcNAc over a longer time period *in vivo*, allowing lower concentrations to be effective at raising *N*-glycan branching. The rate of macropinocytosis may also be much higher *in vivo* compared with *in vitro*.

GlcNAc is also known to be highly safe in humans. In addition to breastfed infants consuming significant quantities, large intravenous doses of GlcNAc (20 g and 100 g) in humans demonstrated no toxicity issues and no alterations in blood glucose or insulin (63, 64). Moreover, treatment with insulin had no effect on the serum *t*_1/2_ of GlcNAc (63). Oral GlcNAc (3–6 g/day) has also been used in 12 children with inflammatory bowel disease for ∼2 years without reported toxicities and/or side effects (65). In rats, chronic systematic toxicological studies at doses of 2323–2545 mg/kg/day for up to 114 weeks found no toxicity (66, 67). Coupled with availability as a dietary supplement, oral GlcNAc may provide a potent, inexpensive, and safe therapy for MS. Large double-blind placebo-controlled trials are warranted to investigate this hypothesis.

## Experimental procedures

### Mouse brain and neural stem cell isolation and analysis

Mice were bred and utilized as approved by the University of California, Irvine Institutional Animal Care and Use Committee. Dorsal forebrain cortical tissue was dissected from the medial ganglionic eminence at embryonic day 12.5 (E12.5) of CD1 mice (Charles River Laboratories) or *Mgat5*^−/−^ C57BL/6 mice and their WT littermates and placed in dissection buffer: PBS, 0.6% glucose, 50 units/ml Pen/Strep. Tissue from multiple embryos within the same litter were pooled, and a subsequent culture from a single litter was considered a biological repeat. The tissue was dissociated using 0.05% trypsin-EDTA at 37 °C for 10 min, followed by treatment with soybean trypsin inhibitor (Life Technologies). Dissociated cells were resuspended in proliferation medium containing DMEM, 1× B27, 1× N2, 1 mm sodium pyruvate, 2 mm l-glutamine, 1 mm *N*-acetylcysteine, 20 ng/ml epidermal growth factor (EGF) (PeproTech), 10 ng/ml basic fibroblast growth factor (bFGF) (PeproTech), and 2 µg/ml heparin, seeded at 150,000 cells/ml (nontissue culture–treated plastic plates), and grown as nonadherent spheres. Cell cultures were passaged approximately every 3 days using enzyme-free NeuroCult Chemical Dissociation Kit (mouse) (Stemcell Technologies). The cultures were passaged at least once prior to experimental use. For experiments, passaged cells were cultured in proliferation media (bFGF and EGF) or differentiation media (bFGF (10 ng/ml) and PDGF-AA (10 ng/ml); Life Technologies) for 48 h with or without the presence of GlcNAc (Ultimate Glucosamine, Wellesley Therapeutics) or kifunensine (GlycoSyn). Neurospheres were dispersed using the enzyme-free NeuroCult kit before being analyzed by flow cytometry using one or more of the following antibodies: anti-CD140a/PDGF-RA PE conjugate (1:200, A15785, Molecular Probes), anti-O4 Alexa Fluor 488 conjugated (1:200, FAB1326G, R&D Systems), anti-GalC Alexa Fluor 647 (1:200, MAB342-AF647, Millipore), and anti-Olig2 (1:200, AB9610, Millipore) with anti-rabbit Alexa Fluor 488 (1:200, Thermo Fisher Scientific).

### GlcNAc treatment of mouse pups

GlcNAc (1 mg/ml) in drinking water was provided to pregnant PL/J mothers or mothers who recently delivered pups and were nursing their young. After the treatment period, pups were anesthetized with isoflurane and cardiac-perfused with PBS. Pup and fetal brains were removed and homogenized by trituration using glass pipettes in PBS with 5% FBS. The cells were then stained with antibodies and analyzed by flow cytometry using antibodies described above. For immunofluorescence analysis of pup brains, pups were quickly decapitated, and brains were harvested and fixed in 4% paraformaldehyde overnight.

### [U^13^C]GlcNAc treatment of mice

[U^13^C]GlcNAc was purchased from Omicron Biochemicals and put in the drinking water at 1 mg/ml of female mice aged 8 weeks for 3 days. Fresh solution of [U^13^C]GlcNAc in drinking water was provided each day. After 3 days, mice were anesthetized with isoflurane and underwent cardiac perfusion with 50 ml of PBS. Brains were harvested and snap frozen in liquid nitrogen. Tissues were cut into 0.04-g pieces and crushed mechanically before undergoing extraction as described below (“Targeted LC–MS/MS”). Levels of UDP-[U13C]GlcNAc were measured by LC–MS/MS analysis as described below (“Targeted LC–MS/MS”).

### Tamoxifen-induced deletion of Mgat1

*Mgat1^f/f^*Plp1-cre/ERTc^+^ and *Mgat1^f/f^*Pdgfra-creER^+^ were generated by crossing our *Mgat1^f/f^* mice with Plp1-cre/ERTc^+^ and Pdgfra-creER^+^ lines from The Jackson Laboratory. Tamoxifen was dissolved in corn oil overnight at 37 °C at a concentration of 20 mg/ml. *Mgat1^f/f^*Plp1-cre/ERTc^+^ and *Mgat1^f/f^*Pdgfra-creER^+^ mice (mean age P71.24, S.D. 1.393) and their control *Mgat1^f/f^* littermates were injected intraperitoneally with tamoxifen (75 mg/kg) daily for 3 days starting on day 0 and sacrificed at 2 weeks or retreated with tamoxifen and sacrificed at 8 weeks. Mice were sacrificed following anesthesia and cardiac perfusion with PBS. Brains examined by flow cytometry were first homogenized by trituration using glass pipettes in PBS with 5% FBS. Brains examined for myelin content were drop-fixed in 4% paraformaldehyde overnight.

### Cuprizone-induced demyelination

Cuprizone at 0.2% induces demyelination in the corpus callosum by 3 weeks, with maximum demyelination at 5–6 weeks (68). 8-week-old C57BL/6 mice purchased from The Jackson Laboratory or 8-week-old *Mgat5*^+/−^ C57BL/6 mice were treated with 0.2% cuprizone (Sigma-Aldrich) mixed into milled rodent chow for 6 weeks for the active phase treatment and 5 weeks for the recovery treatment. During active phase treatment, GlcNAc (1 mg/ml) in drinking water or just drinking water (control) was provided for the last 3 weeks of cuprizone treatment. For the recovery phase treatment, GlcNAc in drinking water or control was provided after cuprizone treatment had been stopped. Mice were anesthetized and underwent cardiac perfusion with 4% paraformaldehyde in PBS or 4% paraformaldehyde plus 0.5% glutaraldehyde in sodium cacodylate buffer for immunofluorescence or electron microscopic analysis, respectively. Brains were then fixed overnight in perfusion solution.

### Accelerated rotarod

One day prior to cuprizone treatment, mice were trained on the rotarod by allowing them to run three 5-min trials at a constant 30 rotations per minute (RPM). Mice then underwent weekly testing during cuprizone and GlcNAc treatment on an accelerating rotarod starting at 4 rpm and increasing to 40 rpm over 5 min. Latency for mice to fall was recorded. If a mouse was not running on the rotarod by holding on for three turns, this was considered a fall. For the active phase treatment, one trial was run every week. For the recovery phase treatment, three trials were run for each mouse each week and latencies were averaged. As expected with cuprizone treatment, performance degraded as treatment progressed. Mice whose performance did not drop below a predetermined threshold (200 s) were not used in analysis.

### Immunofluorescence analysis

For NSC immunofluorescence, whole neurospheres were seeded onto laminin-coated coverslips (Neuvitro) in proliferation medium. After 24 h, proliferation medium was removed and replaced with differentiation medium (same components as proliferation medium but excluding EGF, bFGF, and heparin) to induce differentiation. For analysis of mouse brains, brains were incubated in 30% sucrose for at least 72 h, embedded in optimal cutting temperature compound (Tissue-Tek), frozen for at least 48 h at −80 °C, and then cut at 40 microns on a cryostat. Multiple sections from −1 bregma to −2.5 bregma were then stained with antibodies for MBP (1:100; MAB386, Millipore), Olig2 (1:200; AB9610, Millipore), CC1 (1:100; OP90, Millipore), degraded MBP (48) (1:200, AB5864, Millipore), and APP (1:200; clone 22C11). After overnight incubation with primary antibody, tissues were washed and incubated with secondary antibodies: goat anti-rat Alexa Fluor 488 (1:200, Thermo Fisher Scientific), and goat anti-rabbit TxRd (1:200, Thermo Fisher Scientific). APP is a marker for neuro-axonal damage (69–71), whereas co-staining for CC1 and Olig2 are markers of mature oligodendrocytes (72, 73). To examine the amount of myelin, slices were incubated in FluoroMyelin (1:300; F34651, Thermo Fisher Scientific) for 45 min. Images were acquired on a Keyence fluorescence microscope. Mean fluorescence intensity of the medial corpus callosum was measured using ImageJ.

### EM

Three mice from each treatment group (control and GlcNAc) were selected randomly for EM analysis (before other investigations were performed). Portions of these brains from 0 to −1 bregma were rinsed in 0.1 m cacodylate buffer overnight and again for 15 min the next day. 2 × 1–mm blocks of the corpus callosum were dissected out and postfixed with 1% osmium tetroxide in 0.1 m cacodylate buffer for 1 h, rinsed in double distilled H_2_O, dehydrated in increasing serial dilutions of ethanol (70%, 85%, 95%, and 100% × 2) for 10 min each, put in propylene oxide (intermediate solvent) for 2× 10 min, incubated in propylene oxide/Spurr's resin (1:1 mix) for 1 h, and then incubated in Spurr's resin overnight. The blocks were put in a fresh change of resin in flat embedding molds the next day and polymerized overnight at 60 °C. The blocks were sectioned at 1 µm using a Leica Ultracut UCT ultramicrotome. Floating sections were stained in toluidine blue (1% toluidine blue and 2% sodium borate in double distilled H_2_O) at 60 °C for 3 min., mounted on slides, and cover-slipped. Ultrathin sections were sectioned at 70 nm using a Leica Ultracut UCT ultramicrotome. Sections were mounted on 150 mesh copper grids, stained with uranyl acetate and lead citrate, and viewed using a JEOL 1400 electron microscope. Images were captured using a Gatan digital camera. A blinded rater analyzed images by calculating the g-ratio (ratio of the diameter of the axon excluding the myelin sheath divided by the axon diameter including the myelin sheath) and counting the number of total axons, myelinated axons, dystrophic axons (defined as axon diameter > 0.7 µm), degenerating axons, and paranodes. Degenerating axons were identified as axonal swellings containing more than five clustered dark mitochondria and lysosomes. Paranodes were identified as axons with close proximity of the axolemma with the inner membrane of the myelin sheath with a surrounding cytoplasmic portion of oligodendrocyte.

### MS patient cohort

MS patients were recruited from the neuroimmunology clinical trial unit at the NeuroCure Clinical Research Center, Charité - Universitätsmedizin Berlin (Table S1). Inclusion criteria were based on the 2010 revised McDonald criteria, stable immunomodulatory therapy (relapsing-remitting MS) or no treatment (primary progressive MS and secondary progressive MS). Exclusion criteria were acute relapse and/or corticosteroids within 6 months prior to inclusion. Disease course was determined under strict adherence to the 1996 Lublin criteria (74). Blood draws were fasting. The study was approved by the local ethics committee of Berlin (Landesamt für Gesundheit und Soziales (LAGeSo)). All study participants gave written informed consent. Studies were conducted in conformity with the 1964 Declaration of Helsinki in its currently applicable version.

### MRI

MRI was performed at 1.5 Tesla using three-dimensional T1-weighted magnetization prepared rapid acquisition and multiple gradient echo sequences (MPRAGE; T1w) and axial T2-weighted (T2w) sequences. Images were acquired on a Sonata MRI (Siemens Medical Systems, Erlangen, Germany) with TE 4.38 ms, TR 2,110 ms, TI 1.1 ms, flip angle 15° and isotropic resolutions 1 mm^3^ for T1w, and Multiecho TSE with TE 81 ms, TR 5,780 ms, 150° flip angle, resolution 0.5 × 0.5 × 3 mm, no gap for T2w, or on an Avanto MRI (Siemens Medical Systems, Erlangen, Germany) with TE 3.09 ms, TR 1,900 ms, TI 1.1 ms, flip angle 15° and isotropic resolutions 1 mm^3^ for T1w, and 3D TSE with TE 175 ms, TR 3,000 ms, flip angle 120°, isotropic resolutions 1 mm^3^ for T2w. Conventional spin-echo T1-weighted images (TR 1060 ms, TE 14 ms, 3-mm slice thickness, no gap, and 44 contiguous axial slices) were obtained before and 5 min after injection of 0.1 mmol/kg gadolinium-diethylenetriamine pentaacetic acid (Magnevist, Bayer-Schering, Berlin, Germany).

T2w lesion segmentation was performed as previously described (75) using a semi-automated procedure including image co-registration using FLIRT (FMRIB Software Library, Oxford, UK) and inhomogeneity correction as embedded into the MedX v3.4.3 software package (Sensor Systems Inc., Sterling, VA, USA). Bulk white matter lesion load and lesion count of T2w scans were routinely measured using MedX.

For calculation of T1w/T2w ratio maps, MPRAGE, FLAIR, and T2w scans were reoriented to standard space, bias field corrected, and cropped to a robust field of view using FSL 5.0.9 (76). The MPRAGE and FLAIR scans were then linearly co-registered to T2w using FSL FLIRT and then registered to Montreal Neurological Institute space and brain extracted using the Brain Extraction Toolbox (76). T2w lesions were then automatically segmented by applying the lesion prediction algorithm to FLAIR scans, implemented in the Lesion Segmentation Toolbox version 2.0.15 (77) for Statistical Parametric Mapping (SPM). Gray matter, white matter, and brain masks were then extracted from the MPRAGE. The lesion mask was subtracted from these masks to remove any lesion effects. The T1w/T2w ratio was created by dividing the processed MPRAGE scans by the processed T2w scans. Median T1w/T2w ratios were extracted from the normal-appearing white matter, gray matter, and brain masks.

### Targeted LC–MS/MS

Serum samples for metabolomics analysis were prepared as described previously (78). Briefly, 50 µl of serum (stored at −80 °C) and 200 µl of ice-cold extraction solvent (40% acetonitrile: 40% methanol: 20% H_2_O) were vortexed for 2 min, then shaken in an Eppendorf shaker (Thermomixer R) at 1400 rpm, 4 °C for 1 h and centrifuged at 4 °C for 10 min at ∼18,000 × *g* in an Eppendorf microfuge. Supernatants were transferred to a clean tube and evaporated in a Speedvac (Acid-Resistant CentriVap Vacuum Concentrators, Labconco). Dried samples were stored at −80 °C. The samples were resuspended in 100 µl of water containing the Internal Standards D^7^-Glucose at 0.2 mg/ml and H-tyrosine at 0.02 mg/ml. The samples were resolved by LC–MS/MS in negative mode at the optimum polarity in Multiple reaction monitoring (MRM) mode on an electrospray ionization triple-quadrupole mass spectrometer (AB Sciex 4000 QTRAP, Toronto, Ontario, Canada). MultiQuant software (AB Sciex, Version 2.1) was used for peak analysis and manual peak confirmation. The results, expressed as area ratio (area of analyte/area of internal standard), were exported to Excel and analyzed with MetaboAnalyst 3.0. Standard curves were prepared by adding increasing concentrations of GlcNAc or *N*-acetyl-d-[UL-^13^C_6_]glucosamine ([UL^13^C_6_]GlcNAc) (Omicron Biochemicals, Indiana) to a 50-µl aliquot of control serum. This way we were able to create a calibration curve for HexNAc serum levels, obtaining absolute values rather than relative concentrations. Analysts were blinded in regard to sample origin (healthy control or MS).

### Statistical analysis

Statistical analyses for the *in vitro* and animal experiments were done with Graphpad Prism by *t* tests, with analysis of variance (ANOVA) with Sidak's post-test correction, or by comparing best-fit curves from nonlinear regression (Y = Bmax*X/(*K_d_* + X) as described in the relevant figure legends. Statistical analyses for the clinical part were performed with R Project version 3.5.3. Correlation between serum HexNAc levels and lesion measurements were analyzed using nonparametric Spearman's Rho analyses. Correlations between HexNAc levels and T1w/T2w-ration measurements were analyzed using linear regression models with HexNAc levels as an independent variable.

## Data availability

All data are contained within the manuscript.

## Supplementary Material

Supporting Information
