# Supplementary material for: N-acetylglucosamine drives myelination by triggering oligodendrocyte precursor cell differentiation
Source: J Biol Chem. 2020 Sep 25;295(51):17413–24. doi: 10.1074/jbc.RA120.015595 (PMC7762951; doi:10.1074/jbc.RA120.015595)
Supplement: Supporting Information [file supp_295_51_17413__index.html]

N-Acetylglucosamine drives myelination by triggering oligodendrocyte precursor cell differentiation — N-acetylglucosamine and myelination — N-acetylglucosamine drives myelination by triggering oligodendrocyte precursor cell differentiation — N-acetylglucosamine and myelination — Supporting Information 

# *N*-acetylglucosamine drives myelination by triggering oligodendrocyte precursor cell differentiation

## Supporting Information

- Supporting Information (to be published online) - Figures S1-3, Table S1
