# Supplementary material for: N-acetylglucosamine drives myelination by triggering oligodendrocyte precursor cell differentiation
Source: J Biol Chem. 2020 Sep 25;295(51):17413–24. doi: 10.1074/jbc.RA120.015595 (PMC7762951; doi:10.1074/jbc.RA120.015595)
Supplement: Supporting Information [file supp_RA120.015595_162853_1_supp_602035_qh5vd3.pdf]

Supplementary Materials:

Supplementary Fig 1-3, Supplementary Table S1

Supplementary Figure Legends

Supplementary Figure 1. GlcNAc promotes oligodendrogenesis from precursor cells. (**A**) UDP-GlcNAc, which is synthesized *de novo* from glucose or salvaged from GlcNAc, is the donor substrate utilized by the Mgat branching enzymes. (**B**) Immunofluorescence microscopy of E12.5 NSC's from CD1 mice cultured in growth media (FGF+EGF) ± GlcNAc for 48 hours. (**C-G**) Flow cytometry of C57BL/6 mouse E12.5 NSC's of the indicated genotypes treated in either growth media (FGF+EGF, C), in differentiation media (FGF+PDGF-AA (10ng/mL)) (E-G) or as indicated (D) with/without GlcNAc 80 mM (C-E), kifunensine for 48hrs (F) or doxycycline pre-treatment for 8 days (G). Data are 3 technical replicates per group and representative of 2 experiments. P-values are by one-way ANOVA with Sidaks's multiple comparison test. (*p<0.05, **p<0.01, ***p<0.001, ****p<0.0001).

Supplementary Figure 2. GlcNAc and N-glycan branching promote primary myelination. (**A**) Flow cytometry of brains from PL/J pups whose mothers were treated with GlcNAc (1mg/mL) in drinking water from P5 or E12.5 to P8 (N=5,5 and 2, 11; one sided t-test). (**B**) *Mgat1*$^{f/f}$Plp1-cre/ERTc^{+} mice were injected with or without tamoxifen (75mg/kg) daily for 3 days and brains analyzed by flow cytometry. Data is representative of 3 mice per group. (**C**) *Mgat1*$^{f/f}$Plp1-cre/ERTc^{+} mice (10 weeks old) were treated with tamoxifen at week 0 and 4, sacrificed at week 8 and cerebellums were analyzed by immunofluorescence microscopy for MBP and myelin (fluoromyelin) (N=5 (2 male, 3 female),8 (6 male, 2 female); one sided t-test). Data points represents average fluorescence of area depicted from 2 slices per mouse. (**D-E**) The indicated mice (10 weeks old) were treated with tamoxifen at week 0 and then sacrificed at week 2 and brains were analyzed by immunofluorescence microscopy for MBP and myelin (fluoromyelin) (N=8 (4 male, 4 female), 6 (4 male, 2 female (D), N=8 (4 male, 4 female), 4 (1 male, 3 female) (E), one sided t-test. Data points represent average fluorescence of area highlighted in red in 2 slices per mouse.

Supplementary Figure 3. Oral GlcNAc promotes oligodendrogenesis and re-myelination while limiting axonal injury. (**A**) Per mouse averages of g-ratio, total axons, myelinated axons, paranodes, and dystrophic axons as well number of degenerating axons from electron micrographs of the medial CC of the Mgat5$^{+/-}$ mice from Fig. 3G (N=3,3 (2 male and 1 female per group)) with data obtained blind to treatment conditions and averaged from 2 fields (g-ratio) or 6 fields per mouse (105 µm/field). Degenerating axons highlighted by red arrows and paranodes denoted by blue asterisks. Mice were treated ± GlcNAc for 4 weeks after 5 weeks of cuprizone. P-value by one-sided t-test and chi-square test. Scale bar = 1µm (**B**) Electron micrographs of 10-week old Mgat5$^{+/+}$ and Mgat5$^{+/-}$ littermates were analyzed for number of myelinated axons per field (N=12, 12 fields; 6 fields per mouse, 105 µm/field, one-sided t-test) and g-ratio (N=839 axons over 6 fields). Scale bar = 1µm. (**C**) Fluoromyelin staining of medial CC of Mgat5$^{+/-}$ mice who were treated ± GlcNAc for 6 weeks after 5 weeks of cuprizone starting at 10 weeks of age (N=3 (3 male), 6 (4 male, 2 female); one sided t-test). Data points represent average of 4 slices per mouse.

Supplementary Figure 1

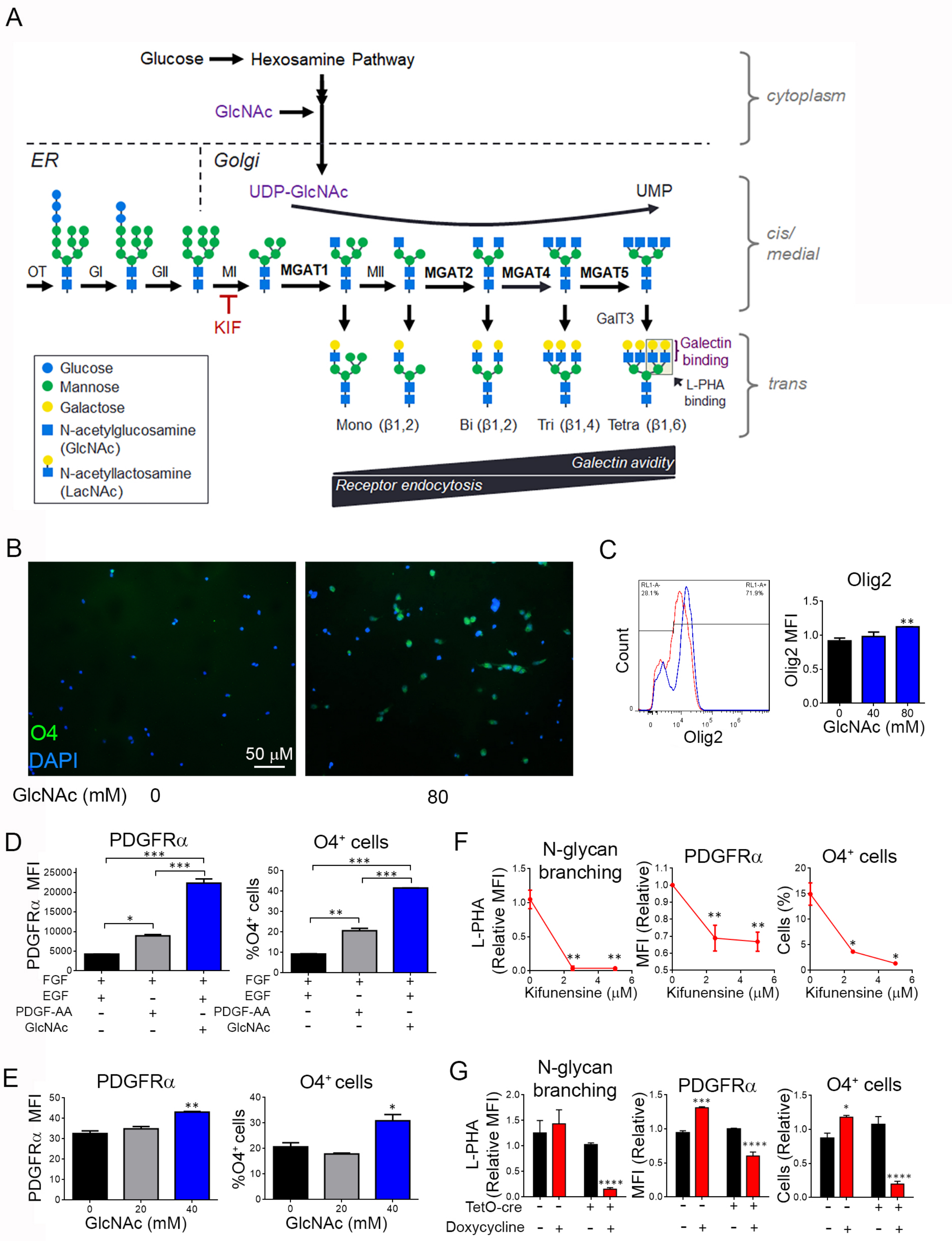

Supplementary Figure 2

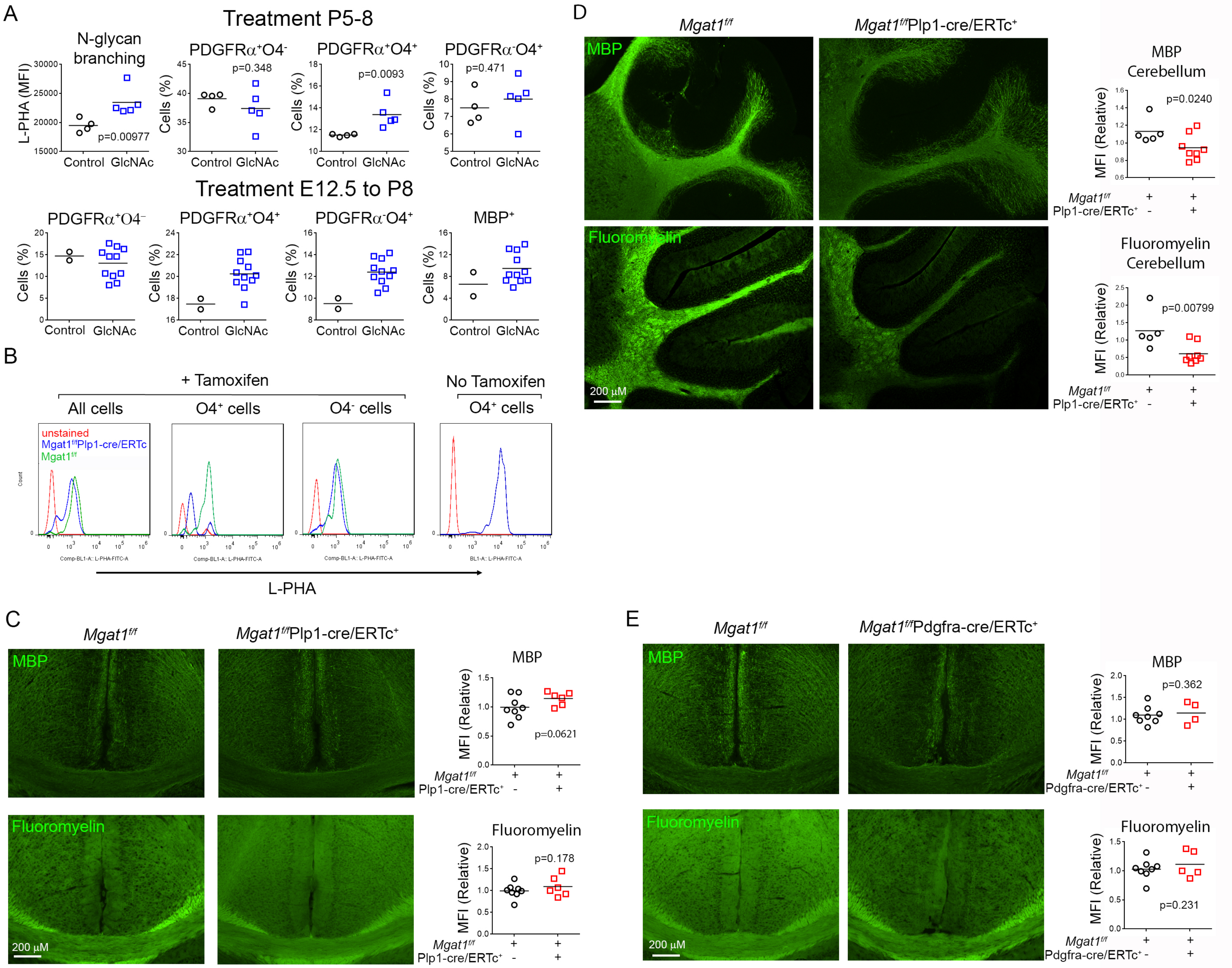

Supplementary Figure 3

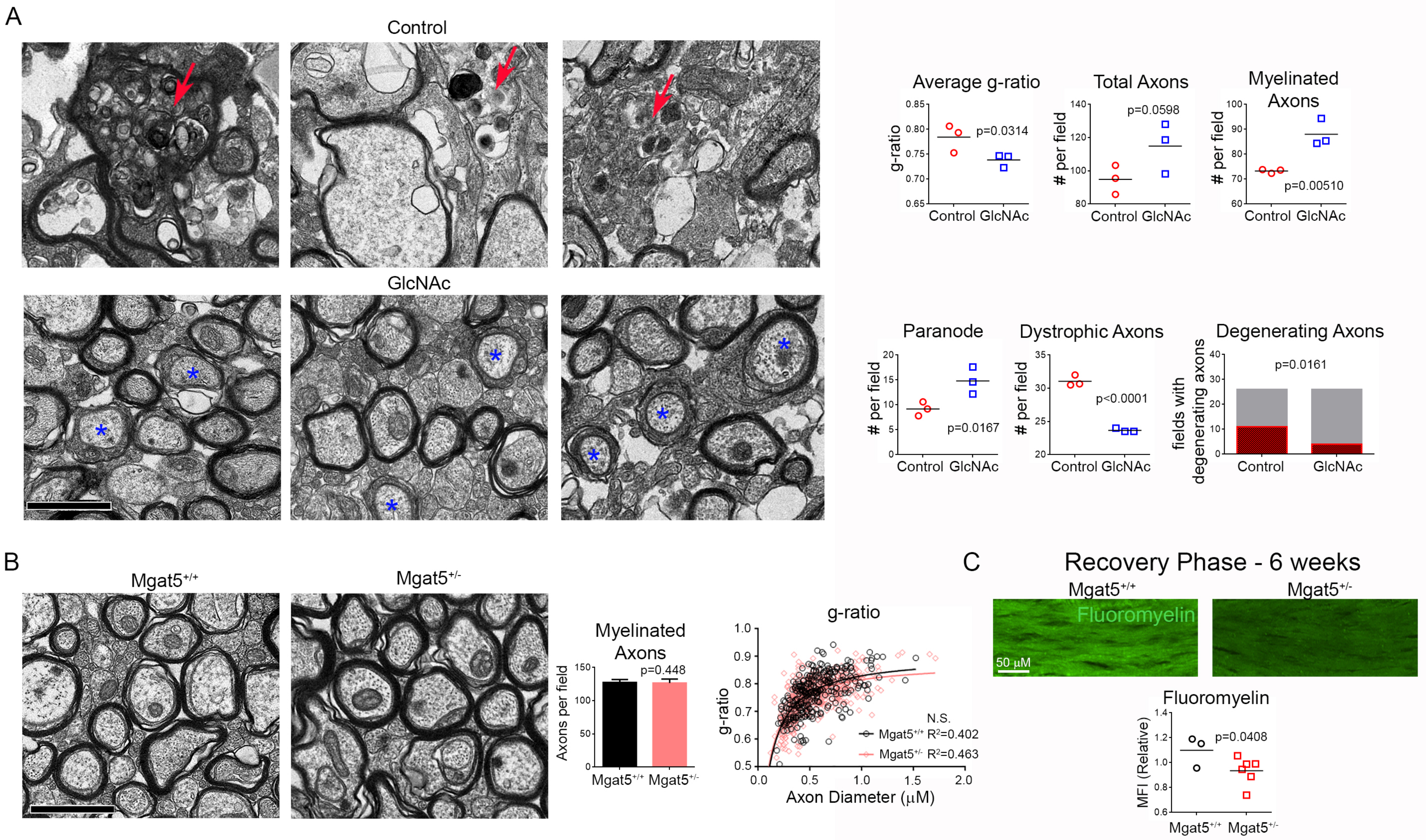

Supplementary Data Table 1 – MS cohort

**MS patients**	*n*	**180**
RRMS	*n*	*125*
PPMS	*n*	*23*
SPMS	*n*	*32*
**Age (years)**	Mean±SD	**42.7±9.4**
**Sex (M/F)**	*n*/*n*	**73/107**
**Time since diagnosis (months)**	Mean±SD	**118.2±87.6**
**EDSS**	Median (Min - Max)	**3.0 (0.0-8.0)**
**T2LC**	Median (Min - Max)	**29 (0 – 162)**
**T2LV (ml)**	Median (Min - Max)	**2790 (0 – 54,900)**
**CELC**	Median (Min - Max)	**0 (0 – 6)**

Abbreviations: MS, multiple sclerosis; RRMS, relapsing-remitting MS; PPMS, primary progressive MS; SPMS, secondary progressive MS; SD, standard deviation; EDSS, Expanded Disability Status Scale; T2LC, T2w lesion count; T2LV, T2w lesion volume; CELC, contrast enhancing lesion count.
